# Water mass mixing controls methane cycling and emission in highly hydrodynamic regions of the open ocean

**DOI:** 10.1093/ismeco/ycaf114

**Published:** 2025-07-10

**Authors:** Xiao-Jun Li, Jinyan Wang, Hao-Nan Wang, Shuang Li, Zhen Zhou, Zhao-Hui Chen, Jiarui Liu, Gui-Ling Zhang, Hong-Hai Zhang, Gui-Peng Yang, Jonathan D Todd, Guang-Chao Zhuang

**Affiliations:** Frontiers Science Center for Deep Ocean Multispheres and Earth System, Key Laboratory of Marine Chemistry Theory and Technology, Ministry of Education, Qingdao 266100, China; Laboratory for Marine Ecology and Environmental Science, Qingdao Marine Science and Technology Center, Qingdao 266100, China; College of Chemistry and Chemical Engineering, Ocean University of China, Qingdao 266100, China; Frontiers Science Center for Deep Ocean Multispheres and Earth System, Key Laboratory of Marine Chemistry Theory and Technology, Ministry of Education, Qingdao 266100, China; Laboratory for Marine Ecology and Environmental Science, Qingdao Marine Science and Technology Center, Qingdao 266100, China; College of Chemistry and Chemical Engineering, Ocean University of China, Qingdao 266100, China; College of Chemistry and Chemical Engineering, Ocean University of China, Qingdao 266100, China; Frontiers Science Center for Deep Ocean Multispheres and Earth System, Key Laboratory of Marine Chemistry Theory and Technology, Ministry of Education, Qingdao 266100, China; Laboratory for Marine Ecology and Environmental Science, Qingdao Marine Science and Technology Center, Qingdao 266100, China; College of Chemistry and Chemical Engineering, Ocean University of China, Qingdao 266100, China; Frontiers Science Center for Deep Ocean Multispheres and Earth System, Key Laboratory of Marine Chemistry Theory and Technology, Ministry of Education, Qingdao 266100, China; Laboratory for Marine Ecology and Environmental Science, Qingdao Marine Science and Technology Center, Qingdao 266100, China; College of Chemistry and Chemical Engineering, Ocean University of China, Qingdao 266100, China; Institute for Advanced Ocean Study, Physical Oceanography Laboratory, Ocean University of China, Qingdao 266100, China; Department of Earth, Planetary and Space Sciences, University of California, Los Angeles, CA 90095, United States; College of Chemistry and Chemical Engineering, Ocean University of China, Qingdao 266100, China; Frontiers Science Center for Deep Ocean Multispheres and Earth System, Key Laboratory of Marine Chemistry Theory and Technology, Ministry of Education, Qingdao 266100, China; Laboratory for Marine Ecology and Environmental Science, Qingdao Marine Science and Technology Center, Qingdao 266100, China; College of Chemistry and Chemical Engineering, Ocean University of China, Qingdao 266100, China; College of Chemistry and Chemical Engineering, Ocean University of China, Qingdao 266100, China; School of Biological Sciences, University of East Anglia, Norwich Research Park, Norwich NR4 7TJ, United Kingdom; Frontiers Science Center for Deep Ocean Multispheres and Earth System, Key Laboratory of Marine Chemistry Theory and Technology, Ministry of Education, Qingdao 266100, China; Laboratory for Marine Ecology and Environmental Science, Qingdao Marine Science and Technology Center, Qingdao 266100, China; College of Chemistry and Chemical Engineering, Ocean University of China, Qingdao 266100, China

**Keywords:** dissolved methane, aerobic methane oxidation (MOx), methane production, water mass mixing, Northwest Pacific

## Abstract

Ocean circulations and water mass exchange can exert significant influences on seawater biogeochemistry, microbial communities, and carbon cycling in marine systems. However, the detailed mechanisms of the impacts of physical processes in the open ocean on the cycle of greenhouse gases, particularly methane, remain poorly understood. In this study, we integrated high-resolution underway observations, experimental incubations, radioisotope labelling, and molecular analysis to constrain the controls of methanogenic pathways, methanotrophic activity, and emission fluxes in the highly hydrodynamic Kuroshio and Oyashio Extension (KOE) region of the Northwest Pacific. The mixing of high-temperature, nutrient-rich Kuroshio waters with methane-rich Oyashio currents significantly affected not only methane abundance, but also methane production pathways and oxidation rates. Water mass mixing caused changes in the dominance of phytoplankton communities to *Bacillariophyta*, with less production of the methane precursor dimethylsulphoniopropionate, thus reducing dimethylsulphoniopropionate-dependent methanogenesis. The alteration of nutrient levels due to mixing of Kuroshio and Oyashio at KOE is also likely to affect microbial utilization of dissolved organic phosphorus, thus influencing methane production from the C−P cleavage of methylphosphonate. Furthermore, the abundances of methanotrophs, such as *Methylocystis* and *Methylosinus*, were much higher at the KOE sites than those observed at the Oyashio Extension, which contributed to elevated methane oxidation rates in the mixing region. Microbial oxidation as a biological sink of methane accounted for ~43.7% ± 28.8% of the total methane loss, which reduced methane emissions to the atmosphere. These data highlight the physical controls on biogeochemical methane cycling, indicating that intensive mixing of water masses may regulate methane emissions from the open oceans.

## Introduction

Methane (CH_4_) is the second most important greenhouse gas after carbon dioxide (CO_2_) and accounts for ~20% of the global warming that has occurred since preindustrial times [1]. Although methane emissions from global oceans are dominated by shallow coastal waters, open ocean waters (depth >2000 m) are slightly oversaturated (0.02-0.2 nmol l^−1^) and also contribute large amounts of methane to the atmosphere (0.6-1.4 Tg y^−1^) owing to the vast open ocean area. Field observations and regression models have revealed that methane oversaturation in the open ocean may be linked to *in situ* methane production [2]. Microorganisms cycle methylated compounds, such as methylphosphonate and dimethylsulphoniopropionate (DMSP), which are thought to contribute to methane production in these oxygenated water [3–5]. A significant proportion of methane in seawater is likely consumed by aerobic methane oxidation (MOx), which prevents the release of methane to the atmosphere [6–8]. Despite the distribution of methane in the oceans being well characterized, little is known regarding the environmental controls of methanogenic and methanotrophic activity in the open ocean. Ocean circulation and water mass exchange may influence methane cycling, but the mechanisms by which physical and biogeochemical processes affect methane production, oxidation, and emissions in the oceanic waters remain unclear [6, 8, 9].

One of the major sinks of atmospheric CO_2_ among global oceans is the Northwest Pacific, where complex advection and mixing processes occur [10–12]. Two major western boundary currents with distinct properties, Kuroshio and Oyashio, converge in the Kuroshio and Oyashio Extension (KOE), making this region unique in seawater chemistry, microbial ecology, and air–sea exchange [13, 14]. The global synthesis of air–sea CO_2_ flux measurements reveals that the KOE region is the major CO_2_ sink for the Earth's oceans [11], which makes the region of the western boundary current as the hotspot for carbon cycling [15]. Previous observations have demonstrated that the KOE is a strong CO_2_ sink with an annual flux of −0.19 Pg C y^−1^, which could account for 10.6% of the global ocean uptake of CO_2_ [16, 17]. Methane and other hydrocarbons were oversaturated relative to the atmosphere in this region and exhibited large amounts of variability due to shifts in the hydrographic conditions [18]. Physical mixing of water mass may change gas chemistry and nutrient availability [19, 20]. The variability in environmental factors such as temperature and nutrients could further control and reshape the phytoplankton structure and microbial community [21, 22]. Together, these physical, chemical, and biological changes exert important influences on methane production pathways, enzymatic activity of oxidation, and eventually methane emission [8, 23]. Given these features, KOE provides an ideal example for investigating the physical and biological controls of greenhouse gas cycling and emissions in the open ocean.

Furthermore, ocean mixing plays a significant role in the climate system and participates in shaping transient climate change (e.g., anthropogenic ocean heat and carbon uptake, sea level rise, and changes in nutrient fluxes) [24]. The oceanic emission of greenhouse gases such as methane could contribute to the atmospheric budget and global warming, while the impact of ocean mixing on methane biogeochemistry in the open ocean has been understudied [24, 25]. In this study, we sought to reveal a detailed mechanism of how water mass mixing affects methane production, oxidation, and emissions in highly hydrodynamic regions of the open ocean. Therefore, we conducted a variety of biogeochemical measurements in the KOE to investigate the control of methane cycling in this carbon sink region of the North Pacific Ocean. We integrated high-resolution observations, experimental incubations and radioisotope labelling and studied methane concentrations, production potential, and methanotrophic activity with environmental variables along two transects across KOE. These data allowed delineation of a detailed mechanism for methane biogeochemical cycling related to water mass properties, implying that ocean mixing is an important control for methane cycling and emissions in the open ocean.

## Materials and methods

### Sample collection

**Figure 1:**
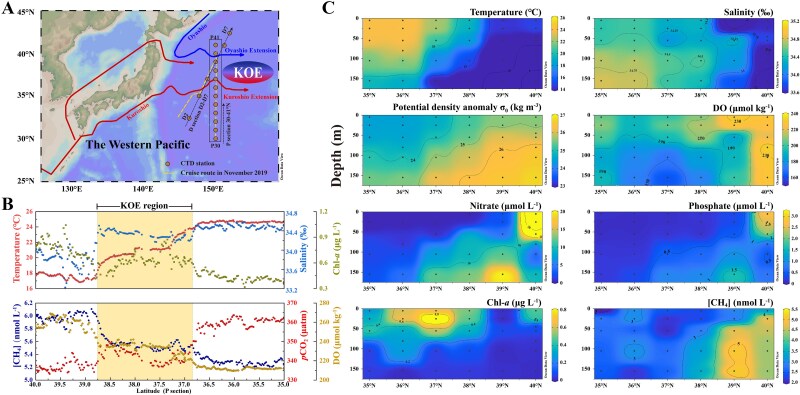
Biogeochemical parameters of the KOE region. (A) The location of the current axis of the Oyashio and Kuroshio are marked with solid lines. The underway investigation routes in November 2019 are plotted with dashed lines. The dots represent the sites of the P transect (150° E) and D transect. (B) The distribution of temperature, salinity, Chl-*a*, DO, and *pCO_2_*, and the concentration of CH_4_ ([CH_4_]) in the P transect surface seawater in November 2019. (C) The vertical distribution of temperature, salinity, potential density anomaly σ_θ_, DO, nitrate, phosphate, Chl-*a* and [CH_4_] at the P transect (35°–40° N) in November 2019.

Seawater samples were collected along the D and P transect across a number of sites in the Northwest Pacific on board the research vessel (R/V) “DongFangHong 3” in 2019, 2021, and 2022 (Fig. 1A and Fig. S1). The D transect refers to the study sites from D1 to D6, while the P transect was located at 150°E spanning sites from 30°N to 41°N. Water samples were collected using Niskin bottles assembled on a Seabird 911-plus conductivity-temperature-depth rosette system (Seabird Corporation, United States). For methane analysis, ~120 ml seawater was gently introduced into brown glass bottles without headspace and then ~50 μl saturated HgCl_2_ solution was added to inhibit biological activities. Samples were sealed with a butyl rubber stopper, crimped with an aluminum cap and stored at 4°C in the dark before analysis. Approximately 1 l seawater was filtered through a 0.7 μm Whatman filter for chlorophyll-*a* (Chl-*a*) measurement. Another 1 l seawater was collected in plastic bottles and preserved with Lugol's solution (final concentration 2%) for the identification of phytoplankton. Nutrient samples (~60 ml) were collected into acid-washed polytetrafluoroethylene bottles and stored at −20°C. For dissolved organic carbon analysis, ~40 ml seawater was filled through pre-combusted 0.45 μm glass fiber filters (450°C, 3 hours) and the filtrate was collected into glass bottles. Subsamples (~4 ml) for DMSP measurements were transferred into 10 ml tubes containing 40 μl 50% H_2_SO_4_ [26, 27]. Dissolved oxygen (DO) was sampled following the Winkler titration method [28].

## CH_4_ concentration and biochemical analyses

Dissolved CH_4_ was measured using a cryogenic purge-and-trap system connected to an Agilent GC-8890 gas chromatographer with a flame ionization detector [7, 29, 30]. Dissolved organic carbon was determined by a nondispersive infrared gas analyzer (Shimadzu TOC-VCPH, Japan) using the high-temperature catalytic oxidation method. Nutrient samples were determined with an AA3 nutrient analyzer (Seal Analytical, United Kingdom). The Chl-*a* was analyzed with a fluorescence spectrophotometer (Hitachi F-4500). Phytoplankton species were identified, and cell numbers were counted using a phytoplankton enumeration chamber under the microscope (Olympus BX51, Olympus Corporation, Tokyo, Japan). The DMSP concentration was measured using a purge-and-trap system connected to gas chromatography with a flame photometric detector as described before [26, 27].

### Continuous measurement of methane and carbon dioxide with an observation system underway

The observations underway along the P and D transect in the Northwest Pacific were conducted on board the R/V *DongFangHong 3* in November 2019 (Fig. 1A). Surface seawater at a depth of 5 m was sampled via a stainless-steel pipeline pump system with a flow rate limited to 1 l min^−1^. The separation of gas and water was achieved using a spraying balancer [29]. The atmospheric sample was collected from the prow ~10 m above the sea surface with a flow rate of 300 ml min^−1^. Because the sensitivity and accuracy of the detector were affected by humidity, moisture was removed with an electric dehumidifier and the chemical desiccant magnesium perchlorate, Mg(ClO_4_)_2_. For CH_4_ and CO_2_ analysis, the sample was filtered with a 0.1 μm membrane and then quantified with a gas concentration analyzer (Picarro G2131-i, United States) using cavity ring-down spectroscopy. The instrument was calibrated every 3 hours using a standard gas mixture (2 ppm CH_4_/200 ppm CO_2_, 4 ppm CH_4_/400 ppm CO_2_, and 6 ppm CH_4_/600 ppm CO_2_, China National Research Center for Certified Reference Materials). The sea surface temperature, salinity, DO, and Chl-*a* were monitored by use of a Ferrybox (4H-JENA, Germany) equipped with multiple probes (SBE45, United States; Seapoint Chlorophyll Fluorometer, United States; Aanderaa Oxygen Optode 4835, Norway).

### Incubation experiment for methane production

**Figure 2:**
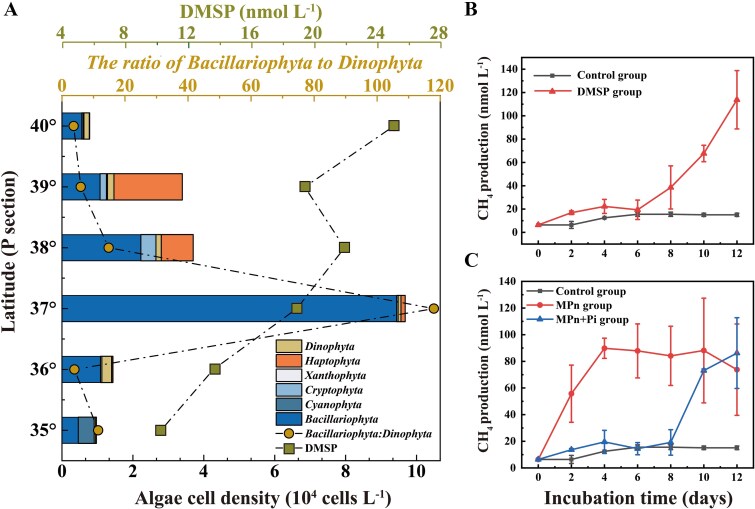
Phytoplankton community composition along the P transect at the KOE and methane production incubation experiments conducted at site D5. (A) The distribution of different phytoplankton communities based on phylum classification (column), DMSP concentration (square) and the ratio of *Bacillariophyta* to *Dinophyta* (dot) of surface water in the P transect in November 2019. (B) and (C) CH_4_ production with the MPn, MPn + pi (MPn and inorganic phosphate) and DMSP amended incubations at *in situ* temperature. The error bars represent the standard deviation from triplicates.

Incubation experiments were conducted to assess the methane production pathway from MPn and DMSP at site D5 during the 2022 cruise (Fig. 2). Briefly, ~400 ml surface water was transferred into 500 ml glass bottles, which were then amended with MPn or DMSP. Two different treatments were amended for the MPn group: (i) MPn was added to a final concentration of 1 μmol l^−1^ or (ii) 0.5 μmol l^−1^ MPn and 0.5 μmol l^−1^ dissolved inorganic phosphorus (Pi) were added as the MPn + Pi group. The DMSP group was amended with 10 μmol l^−1^ DMSP. Each treatment was conducted in triplicate and a control without amendment of MPn or DMSP was processed identically to the treatment group.

The bottles were sealed with a butyl rubber plug and screw cap, and two rubber tubes (one long and one short) were securely connected to the plug for sampling. All samples were incubated at *in situ* temperature in the dark. Seawater samples were obtained using a gastight syringe through introducing the same volume of air at different time points and methane concentrations were quantified with a gas chromatography as described above. Given the complexity and variability of natural water bodies, the microcosm experiments were not identical to the *in situ* conditions but could provide general information for the methane production pathways.

### Measurement of MOx rate

The MOx rate was quantified with a radiotracer approach using ^3^H-labeled methane [7, 30, 31]. Briefly, approximately 20 ml of seawater was added to 4 Hungate tubes without headspace (including triplicate samples and a killed control) and then capped with a butyl rubber stopper. Then, an ~50 μl aliquot of ^3^H-CH_4_ gas (>10^5^ disintegrations per minute (DPM); American Radiolabeled Chemical, Inc.) was injected into each replicate by displacing the same volume of seawater. To terminate the activity of control samples, 2.4 ml of 37% formalin was amended before tracer additions. Samples were incubated in the dark at *in situ* temperature for 48 hours, and the product of the oxidation (^3^H-H_2_O) was quantified after incubation separately. After incubation, ~100 μl subsample was collected into 2 ml vials filled with 1.7 ml scintillation cocktail, and the sample was counted for the total radioactivity (^3^H-H_2_O and ^3^H-CH_4_). The remaining samples were collected into 45 ml tubes containing 2.4 ml 37% formaldehyde and purged with nitrogen gas for 1 hour to remove the dissolved ^3^H-CH_4_, then ~2 ml sample was dispensed into a 6-ml scintillation vial containing 4 ml scintillation cocktail and counted for the product of the oxidation (^3^H-H_2_O). MOx rates were calculated using the following equations according to the first-order kinetics:


(1) \begin{equation*} k=\frac{DPM_{\left[{}^3H-{H}_2O\right]}}{DPM_{\left[{}^3H-{H}_2O+{}^3H-C{H}_4\right]}}\bullet \frac{1}{t} \end{equation*}

(2) \begin{equation*} MOx=k\bullet{\left[C{H}_4\right]}_{in\ situ}, \end{equation*}

where *k* is the first-order rate constant and [CH_4_]_*in situ*_ is the methane concentration measured in separate vials. Furthermore, bacterial production rates were measured with ^3^H labelled leucine (American Radiolabeled Chemical, Inc.). The details of this approach were described previously [14, 32].

### Microbial community and gene quantification

To investigate the methanotrophic community, we collected seawater samples from three sites (D5, P40, and P41). We filtered ~2 l seawater through 0.22 μm polycarbonate membranes (GTTP, 142 mm, Millipore) and stored the filters at −80°C before analysis. DNA was extracted using a DNeasy® PowerSoil® Pro Kit (QIAGEN, United States) and following the protocol of the manufacture. Primers A189F (5′-GGNGACTGGGACTTCTGG-3′) and mb661R (5′-CCGGMGCAACGTCYTTACC-3′) were used to amplify the bacterial *pmoA* gene. Sequencing was performed on an Illumina MiSeq PE300 platform (Illumina, San Diego, United States) according to the standard protocols (Majorbio Bio-Pharm Technology Co. Ltd., Shanghai, China). The taxonomy of each OTU representative sequence was analyzed by use of RDP Classifier version 2.2 against the function gene database (fgr/pmoA_2012) using a confidence threshold of 0.7.

The quantitative polymerase chain reaction (qPCR) analyses were conducted using an Applied Biosystems QuantStudio 5 instrument and analyzed with QuantStudio™ Design & Analysis Software version 1.5.2. Each reaction had a total volume of 20 μl, comprising 10 μl of SYBR Premix Ex Taq™ II (Takara, China), 0.4 μl of each primer, 1 μl of template DNA, and 8.2 μl of double-distilled H_2_O. The thermal cycling program included an initial denaturation step at 95°C for 30 seconds, followed by 40 cycles of denaturation at 95°C for 25 seconds, annealing at 53°C for 30 seconds, and extension at 72°C for 30 seconds. A quantification standard was established using a series of 10-fold diluted purified plasmid DNA derived from the cloned genes isolated from sediment samples, with all standard curves exhibiting *R^2^* values exceeding 0.99. The primers used for the bacterial 16S rRNA gene amplification were 338F (5′-TCCTACGGGAGGCAGCAGT-3′) and 806R (5′-GGACTACCAGGGTCTAATCCTGTT-3′), while the primers for the *pmoA* gene were designed similarly to those used in community analysis of methane-oxidizing bacteria.

### Optimum multiparameter analysis

Optimum multiparameter (OMP) analysis was conducted using hydrographic properties to describe the mixing ratios of water masses [33]. The OMP method resolves a system of property conservation equations (Eq. 3) by considering the six properties of water mass: potential temperature (*T*), salinity (*S*), dissolved oxygen (*O*), phosphate (*P*), nitrate (*N*), and silicate (*Si*).


(3) \begin{equation*} \left\{\begin{array}{@{}l}{x}_1\bullet{T}_1+{x}_2\bullet{T}_1+{x}_3\bullet{T}_3+{x}_4\bullet{T}_4+{x}_5\bullet{T}_5+{x}_6\bullet{T}_6={T}_{obs}+{R}_T\\{}{x}_1\bullet{S}_1+{x}_2\bullet{S}_2+{x}_3\bullet{S}_3+{x}_4\bullet{S}_4+{x}_5\bullet{S}_5+{x}_6\bullet{S}_6={S}_{obs}+{R}_S\\{}{x}_1\bullet{O}_1+{x}_2\bullet{O}_2+{x}_3\bullet{O}_3+{x}_4\bullet{O}_4+{x}_5\bullet{O}_5+{x}_6\bullet{O}_6={O}_{obs}+{R}_O\\{}{x}_1\bullet{P}_1+{x}_2\bullet{P}_2+{x}_3\bullet{P}_3+{x}_4\bullet{P}_4+{x}_5\bullet{P}_5+{x}_6\bullet{P}_6={P}_{obs}+{R}_P\\{}{x}_1\!\bullet\! {N}_1+{x}_2\bullet{N}_2+{x}_3\bullet{N}_3+{x}_4\bullet{N}_4+{x}_5\bullet{N}_5+{x}_6\bullet{N}_6={N}_{obs}+{R}_N\\{}{x}_1\! \bullet\! {Si}_1\! +\! {x}_2\!\bullet\! {Si}_2+{x}_3\bullet{Si}_3+{x}_4\bullet{Si}_4+{x}_5\bullet{Si}_5+{x}_6\bullet{Si}_6={Si}_{obs}+{R}_{Si}\\{}{x}_1+{x}_2+{x}_3+{x}_4+{x}_5+{x}_6=1+{R}_M\end{array}\right. \end{equation*}

where *x_i_* represents the contribution (%) of each water mass. *T_i_*, *S_i_*, *O_i_*, *P_i_*, *N_i_*, and *Si_i_* are the parameter definitions for the source water mass. *R_i_* represents residuals, while *T_obs_ S_obs_*, *O_obs_*, *P_obs_*, *N_obs_*, and *Si_obs_* refer to observed properties. The study area is mainly influenced by three water masses: the Kuroshio Extension, Oyashio Extension (OE), and North Pacific Intermediate Water water mass [34]. By examining the Temperature–Salinity diagram, we determined the values of the six properties for each water mass, which are presented in Table S1.

### The calculation of *pCO_2_* and the air-sea fluxes of CO_2_ and CH_4_

The mole fraction of CO_2_ measured by the instrument was converted into *pCO_2_* by use of the following equation:


(4) \begin{equation*} pC{O_2}_{(eq)}=\left[ xC{O}_2/\left(1-x{H}_2O\right)\right]\times \left({P}_{eq}-f\right), \end{equation*}

where *pCO_2(eq)_* is the partial pressure of CO_2_ in the headspace of the equilibrator, *xCO_2_* and *xH_2_O* denote the volume ratios of carbon dioxide and water in the headspace gas, and *P_eq_* is the *in situ* atmospheric pressure. The coefficient (*ƒ*) was the saturated water vapor pressure at 100% humidity, which can be calculated by salinity and temperature (*T_eq_*, Kelvin) at equilibrium (Eq. 5) [35].


(5) \begin{equation*} \mathit{\ln}f=24.4543-6745.09/{T}_{eq}-4.8489\times \mathit{\ln}\left({T}_{eq}/100\right)-0.000544\times S \end{equation*}

The temperature was corrected based on the *in situ* temperature measured by the temperature sensor at the inlet (Eq. 6) [14].


(6) \begin{equation*} pC{O}_2(water)= pC{O}_2(eq)\times \mathit{\exp}\left[0.0423\times \left( SST-{T}_{eq}\right)\right] \end{equation*}

The air–sea fluxes of CH_4_ and CO_2_ were calculated by the difference between surface seawater and atmospheric concentration (Eqs. 7 and 8):


(7) \begin{equation*} {F}_{CH_4}={k}_{C{H}_4}\times \left({\left[C{H}_4\right]}_{water}-{\left[C{H}_4\right]}_{atm}\right) \end{equation*}

(8) \begin{equation*} {F}_{CO_2}={k}_{C{O}_2}\times{K}_H\times \Delta pC{O}_2 \end{equation*}

where *K_H_* is the solubility of CO_2_ in the seawater. In addition, the gas transfer velocity, *k,* was estimated by an empirical formula (Eq. 9) proposed by Wanninkhof [36]:


(9) \begin{equation*} k=0.251\times{u}^2\times{\left( Sc/660\right)}^{-0.5}, \end{equation*}

where *Sc* is the Schmidt number in seawater and *u* is the wind speed at 10 m height, which is recorded by meteorological sensors.

## Results and discussion

### Physical mixing of methane from different water masses

Intensive mixing of the Kuroshio and Oyashio waters occurred at the KOE region from 35° to 40° N (Fig. 1A). The two distinct water masses exhibited significant meridional variations in surface temperature (16.8 to 30.2°C) and salinity (33.2 to 35.1) along the P transect (Fig. 1B). The concentrations of Chl-*a* and DO generally decreased from 40° N to 35° N (Fig. 1B). Likewise, nutrient concentrations such as nitrate and phosphate were much higher in the water columns of Oyashio (>38° N) than those in Kuroshio (<36° N) (Fig. 1C). Underway analysis of dissolved CO_2_ and CH_4_ showed contrasting patterns from north to south (Fig. 1C). While *pCO_2_* increased from ~338 μatm at 38°−40° N to 360 μatm at ~35° N, the concentrations of dissolved CH_4_ decreased from 6.11 to 5.15 nmol l^−1^. Generally, the solubility of dissolved gases in the seawater was influenced by the physical properties of the water mass, such as temperature and salinity. High temperature decreased the solubility of dissolved gases, as observed by the strong negative correlation between CH_4_ and temperature. Multiple linear regression analysis revealed that salinity, temperature, and oxygen saturation significantly influenced the methane concentration (adjusted *R^2^ *= 0.97; *P < *0.01; Table S2). Temperature and salinity accounted for 42.26% and 33.89% of methane variability, respectively. However, the reduced surface *pCO_2_* in cold Oyashio waters indicated that other processes such as biological activity through photosynthesis controlled the distribution of *pCO_2_*, as reflected by the elevated Chl-*a* and DO concentrations. Furthermore, physical mixing of Kuroshio and Oyashio with different concentrations of methane would lead to a dilution of methane with increasing salinity (Fig. S2) [18, 34]. However, the distribution of methane along the transect did not follow an ideal dilution pattern (**r* =* 0.75; *P <* 0.01; Fig. S2). The values below the theoretical dilution line between 35° and 40° N suggested that other biogeochemical processes such as methane production or oxidation might influence methane abundance in the KOE region.

### Methane production modulated by water mass mixing

Methane in open ocean surface waters is largely thought to result from *in situ* production [18]. Aerobic methane production from methylated compounds such as MPn and DMSP has been recognized as the dominant methanogenic pathway in these oxygenated waters [5, 23, 37]. Indeed, the addition of 10 μM DMSP to surface waters stimulated the production of 107 nM methane over 12 days of incubation (Fig. 2B), implying that methane was produced from this methylated sulfur compound. Consistent with this observation, DMSP was detected at relatively high concentrations (10.3-25.1 nM) in the KOE region (Fig. 2A). Interestingly, DMSP was more abundant in Oyashio waters and the concentrations generally decreased from 40° to 35° N. DMSP concentrations did not change with the abundance of phytoplankton, which increased from 7.80 × 10^3^ at 40° to a maximum of 9.68 × 10^4^ cell l^−1^ at 37° N (Fig. 2A and Table S3). This finding could be attributable to the levels of DMSP accumulated by phytoplankton, being species-dependent, with *Dinophyta* and *Haptophyta* regarded as high (intracellular DMSP>50 mM) but *Bacillariophyta* as low (<50 mM) accumulators [38–40]. The north sites contained phytoplankton communities with relatively high levels of *Phaeocystis*, a *Haptophyta* known to accumulate high DMSP levels that likely contributed to the higher DMSP levels at these sites. In contrast, *Bacillariophyta* dominated the phytoplankton communities in the Kuroshio and its extension waters with lower DMSP levels. This shift of phytoplankton communities during the mixing of Kuroshio and Oyashio likely impacts DMSP availability and DMSP-dependent methane production.

Similar to DMSP, methane production was observed with the addition of MPn or MPn + Pi to Kuroshio surface seawater. While methane concentration increased immediately after the amendment of MPn solely, methane was elevated after 8 days of incubation in the MPn + Pi treatment (Fig. 2C). This lag for methane production with Pi suggested that methane was produced from MPn after Pi was consumed. In the oligotrophic ocean, microbes like *Vibrio* are capable of degrading dissolved organic P such as MPn to cope with P-limited conditions, resulting in methane production from the decomposition of dissolved organic phosphoruscontaining C−P bonds [7, 23, 41]. In our study area, nutrient concentrations varied largely between sites from 35° to 40° N. Therefore, methane production through C−P lyase phosphonate degradation might also be impacted by nutrient changes during water mass mixing. The nutrient levels were extremely low in the Kuroshio waters, and MPn degradation could be important for methane production at these sites. Consistently, *Trichodesmium* was relatively abundant at the Kuroshio waters (site P35) with deficient nutrients (Table S3), and the key enzyme of phosphonate synthesis, phosphoenolpyruvate mutase (PepM), can be synthesized in these microorganisms [42, 43]. Previous studies showed that *Trichodesmium* was sensitive to Pi availability and preferentially utilized Pi in the presence of both Pi and dissolved organic phosphorus [43–45]. Abundant nutrient intrusion from the north would relieve the P stress conditions in the mixing region and might impact the utilization of phosphonate compounds, which would further influence the production of methane from C−P lyase. Therefore, water mass exchange not only drives the physical mixing of gases and nutrients, but also influences the phytoplankton communities and further regulates the methanogenesis in the KOE region.

### Differences in methanotrophic activity between water masses

**Figure 3:**
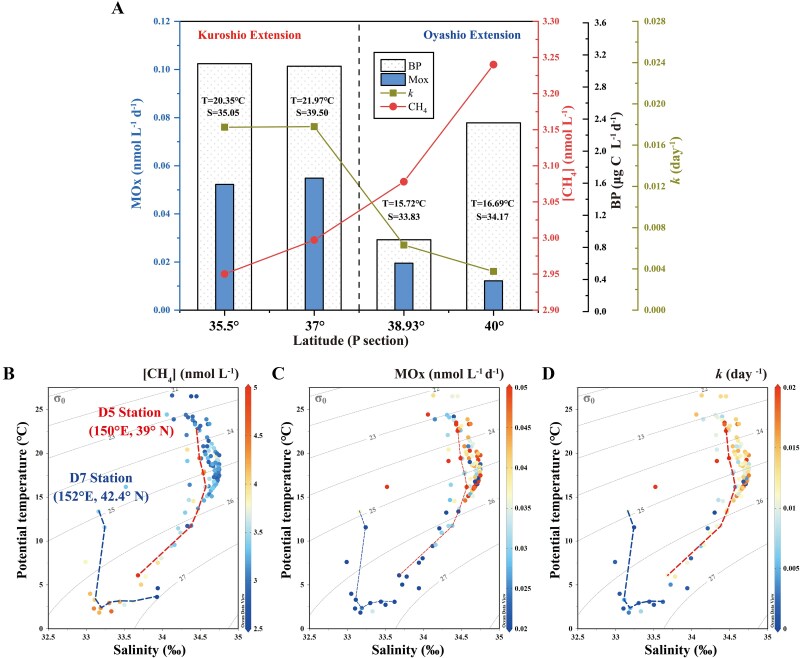
Methane oxidation rates at the KOE region. (A) Bacterial production rate (BP, scatter filled column), methane concentration ([CH_4_], dot), methane oxidation rate (MOx, solid column) and rate constant (*k*, square) in surface seawater at P transect in June 2021. (B–D) Potential temperature versus salinity profile for the P transect in July 2022. The color bar represents the [CH_4_], MOx and rate constant *k*, respectively.

Methane concentrations in surface waters are a consequence of production, oxidation, advection, and/or ventilation rates. MOx is one of the dominant sinks which removes a large fraction of methane produced [7]. A variety of environmental factors have previously been shown to influence methanotrophic activity, e.g., methane concentration, temperature, and nutrients [30, 46, 47]. Particularly, hydrological processes can significantly affect the efficiency of MOx since water masses with distinct oceanographic parameters can influence microbial abundance and activity [8]. To examine the controls of methanotrophic activity in the KOE region, we measured MOx rates with ^3^H-labeled radiotracers. Apparently, MOx rates were much higher at the south sites (e.g., 35°-39° N, 0.046 ± 0.025 nmol l^−1^ d^−1^) than those north sites (e.g., 40°-42° N, 0.027 ± 0.025 nmol l^−1^ d^−1^) during the 2021 expedition (Fig. 3A and Fig. S1). Although methane concentrations were lower, the rate constants *k* at KOE sites were 3.5 times of those at Oyashio sites. The rate constant *k* is usually used as a proxy for methanotrophic biomass which can reflect the instantaneous capacity for methane consumption at low concentrations. Indeed, 16S rRNA amplicon sequencing revealed that diverse Type II methanotrophs belonging to *Alphaproteobacteria* (19.23% relative abundance) were very abundant within KOE (site D5) samples (Table 1). These included the facultative methanotrophs genera of *Methylocystis* and *Methylosinus* which accounted for 5.61% and 3.05% of the bacteria, respectively. It has been reported that Type II methanotrophs display adaptability to diverse conditions, including nutrient limitation [48]. For example, a recent study demonstrated that *Methylosinus trichosporium* OB3b, can produce P-free glycolipids to replace membrane phospholipids in response to P limitation [49]. Therefore, the increased abundance of *Methylocystis* and *Methylosinus* might reflect their adaption to the change of nutrient levels. In contrast, much less bacteria (~0.73% of *Alphaproteobacteria* and ~0.07% of *Gammaproteobacteria*) were predicted to possess *pmoA* at station P40. Furthermore, qPCR analysis also showed markedly elevated copy numbers of the bacterial 16S rRNA and *pmoA* (encoding particulate methane monooxygenase) genes in the upper water columns of site D5 compared to site P40 (Fig. S3). Therefore, it is likely that the difference in methanotroph abundance and community contributed to the observed variation of MOx rates in the Kuroshio and Oyashio waters.

**Table 1:** Comparison of density anomaly (σ_θ_), bacterial productivity (BP), methane concentration ([CH_4_]), methane oxidation rates (MOx rates), and methane oxidation constant (*k*) between different water masses in the upper water column (above 300 m) in the KOE region.^a^

	Oyashio		KOE	Kuroshio
σ_θ_ (kg m^−3^)	26.49 ± 0.23		24.08 ± 0.73	25.36 ± 0.22
BP (μg C l^−1^ d^−1^)	7.08 ± 9.14		8.85 ± 4.86	10.12 ± 8.56
[CH_4_] (nmol l^−1^)	3.77 ± 0.63		3.21 ± 0.40	3.29 ± 0.25
MOx rates (nmol l^−1^ d^−1^)	0.021 ± 0.016		0.044 ± 0.018	0.036 ± 0.015
*k* (d^−1^)	0.006 ± 0.004		0.013 ± 0.005	0.011 ± 0.005
	P41	P40		D5
*Alphaproteobacteria* (%)	ND	0.73		19.23
*Gammaproteobacteria* (%)	ND	0.07		ND

^a^The community composition of methanotrophic bacteria was analyzed at stations P41 and P40, which are influenced by the Oyashio, as well as at station D5, influenced by the Kuroshio. ND, not detected.

Similar to MOx rates, bacterial production rates, which ranged from 0.049 to 8.03 μg C l^−1^ d^−1^, were also generally higher in the Kuroshio than the subarctic region (Fig. 3A and Fig. S1), indicating elevated heterotrophic activity in the Kuroshio. Assuming that the methanotrophs incorporate ~25% of methane carbon into biomass [50], the MOx rates would account for 1.43% of the BP. Furthermore, strong correlations were observed between MOx rates and physical properties such as temperature, density, or salinity (MOx vs temperature, *r* = 0.49; MOx vs σ_θ_, *r *= 0.40; MOx vs current velocity, *r *= 0.38; *P* < 0.01; Fig. S4), further suggesting the controls of water mass in methanotrophic activity. Clearly, both the rate constant *k* and MOx rates can be classified by water masses with salinity and temperature. In addition, two contrasting sites of D5 and D7 located at the KOE and OE, respectively, also exhibited distinct MOx rates, and much higher rates were observed at D5 than D7 (Fig. 3B–D). While no significant relationship was observed between methane concentrations and MOx rates (*P* > 0.05; Fig. S4), the rate constant *k* that reflected enzymatic activity correlated well with the MOx rates (*r* = 0.93; *P* < 0.01). These results indicated that the effect of water mass exchange on the diversity and activity of the methanotrophic community drove the observed variation of MOx rates and that the elevated methanotrophic activity in the mixing region would lead to the rapid biological turnover of methane and maintain methane concentrations at low levels.

### Methane emissions regulated by physical–biogeochemical feedback

**Figure 4:**
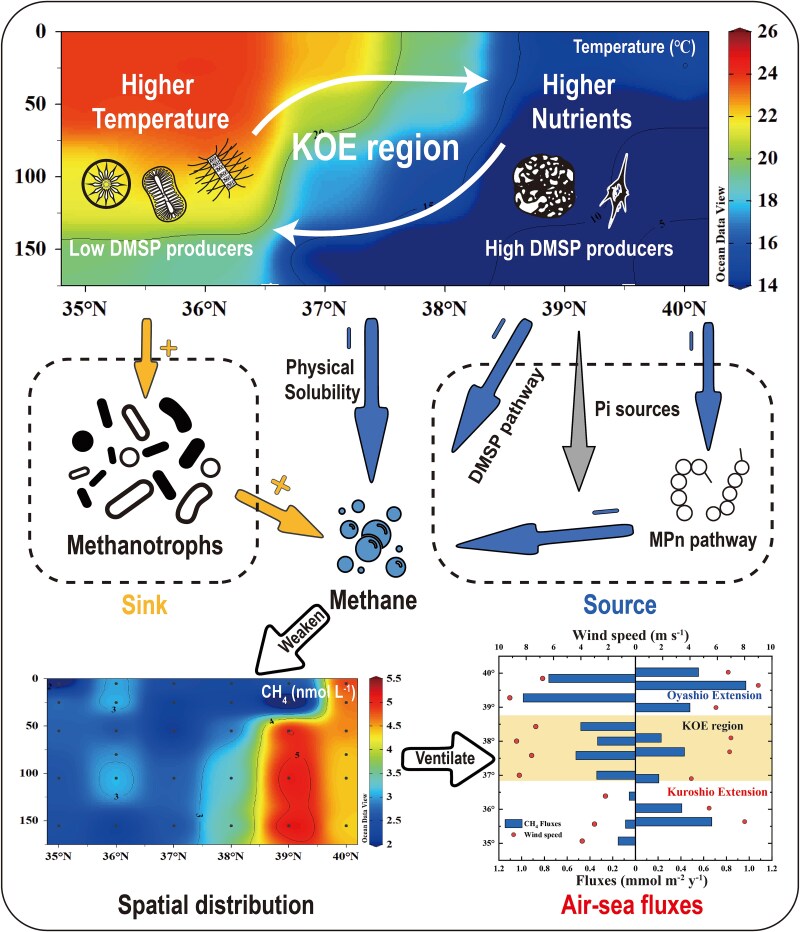
Schematic diagram of the mechanism that controls CH_4_ cycling in the mixing region of KOE. Increased water temperature decreased methane solubility. The dramatic decrease of the abundance of potential DMSP-producing phytoplankton and the inhibition of the methanogenic pathway of methyl compounds degradation by MPn also weakens methane production. Finally, the enhanced activity of methane-oxidizing bacteria accelerated the methane consumption. This mechanism controls the spatial distribution of methane and further influences its air-sea fluxes, which will eventually affect the global climate.

Ultimately, the physical–biogeochemical controls of methane production and oxidation in the mixing region determine the saturation of methane in surface water, which could be linked to the regional emissions of methane into the atmosphere. Water mass mixing significantly influences carbon biogeochemical processes and further impacts air–sea exchange fluxes of greenhouse gases. The Northwest Pacific acts as an important CO_2_ sink (air–sea fluxes: −2.94 ± 2.78 mol m^−2^ y^−1^; ∆*pCO_2_*: −55.3 ± 12.7 μatm), and the air–sea exchange processes were mainly controlled by physical transportation of water masses and phytoplankton activity [10, 11, 51]. In contrast, methane in surface water of the Northwest Pacific was predominantly oversaturated (107.9% ± 2.8%) with respect to atmospheric CH_4_, suggesting that these open waters were a source of atmospheric CH_4_ [18]. Air–sea exchange fluxes of CH_4_ ranged from 0.06 to 0.99 mmol m^−2^ y^−1^ in our study area of the Northwest Pacific (Fig. 4). Apparently, stronger emissions of methane were observed in the OE region than the KOE region. While the wind speed, one of the key factors for flux estimation, varied slightly (7.31 ± 1.38 m s^−1^) between sites at 37°−40° N, the methane concentration gradient dominated the significant differences of the air–sea fluxes. Microbial consumption represented another important sink of methane, and a large fraction of methane could be filtered through aerobic oxidation of methane before ventilation [7, 9, 30]. In the KOE region, enhanced methanotrophic activity resulted in an elevated contribution (43.7% ± 28.8%) of MOx to the total methane removal (the sum of depth-integrated MOx rates and air–sea fluxes), further reducing methane emissions to the atmosphere (Fig. S5). As a result, intensive mixing of water masses not only facilitates carbon storage but also mitigates methane emissions in the Northwest Pacific, suggesting the physical controls on biogeochemical cycling of greenhouse gases. Similarly, a previous study demonstrated that water mass exchange could rapidly reduce methanotrophic activity in the water column above seeps, thus impacting methane cycling in the upper ocean [8]. Other physical processes such as mesoscale eddies could influence methane emissions through the regulation of nutrient replenishment that changed the distribution of phytoplankton communities and methane precursors [29]. Upwellings could bring methane-depleted waters to the surface and decrease surface water methane concentrations as observed in off-shelf waters of the Southern Ocean [52]. Therefore, physical processes in dynamic marine environments could have significant impacts on methane production and consumption pathways and activities, and the balance of production and consumption determine methane concentrations and saturation in surface water, which further drive methane emission fluxes to the atmosphere. Taking all these observations together, given the ubiquity and complexity of ocean circulation and mesoscale in the ocean, the global significance of physical processes on greenhouse gases emission and potential climate effect should be considered and required further investigations.

However, although our study provides a mechanism of how water mixing influence methane cycling in the open ocean, it remains challenging to quantify this impact on methane emission fluxes in both regional and global scales, particularly considering the diversity and variability of ocean currents and multi-scale dynamical processes. Future studies should combine high-resolution field observations and machine learning models to better estimate the effect of physical processes on marine methane emissions in highly hydrodynamic regions of the open ocean, which would advance our current understanding of methane cycling in marine environments.

## Conclusions

In this study, we investigated the controls of methane cycling in the Northwest Pacific Ocean and demonstrated a detailed mechanism of methane biogeochemistry in relation to water mass mixing in the open ocean. We found that the mixing of two distinct ocean currents, the high salinity and temperature and low-nutrient Kuroshio with the low-temperature nutrient-rich Oyashio exert significant influences on methane concentrations, phytoplankton structure, methanotrophic communities, methane production pathways, and MOx activity at the KOE region (Fig. 4). The physical biogeochemical regulation of methane cycling leads to reduced saturations of methane in surface water, which impact regional methane emissions to the atmosphere. Our results highlighted the role of water mixing in controlling methane cycling in the open ocean and have important implications for global oceanic methane emissions. Future investigations are required to further quantify the effect of physical processes on marine methane emission and potential uncertainties, possibly through high-resolution field observations and machine-learning models.

## Supplementary Material

Supplement_material_ycaf114

Supplment_Table_S3_ycaf114

Supplement_Table_S4_ycaf114

## Data Availability

The raw reads were deposited into the NCBI Sequence Read Archive (SRA) database (Accession Number: PRJNA1003286). MOx rates are provided in Supplement Table S4.
