# Supplementary material for: Water mass mixing controls methane cycling and emission in highly hydrodynamic regions of the open ocean
Source: ISME Commun. 2025 Jul 10;5(1):ycaf114. doi: 10.1093/ismeco/ycaf114 (PMC12342928; doi:10.1093/ismeco/ycaf114)
Supplement: Supplement_material_ycaf114 [file supplement_material_ycaf114.pdf]

Supporting Information

Water mass mixing controls methane cycling and emission in highly hydrodynamic regions of the open ocean

Xiao-Jun Li[1, 2, 3], Jinyan Wang[1, 2, 3], Hao-Nan Wang[3], Shuang Li[1, 2, 3], Zhen Zhou[1, 2, 3], Zhao-Hui Chen[4], Jiarui Liu[5], Gui-Ling Zhang[3], Hong-Hai Zhang[1, 2, 3, *], Gui-Peng Yang[3], Jonathan Todd[6], Guang-Chao Zhuang[1, 2, 3*]

[1]*Frontiers Science Center for Deep Ocean Multispheres and Earth System, Key Laboratory of Marine Chemistry Theory and Technology, Ministry of Education, Qingdao, 266100, China.*

[2]*Laboratory for Marine Ecology and Environmental Science, Qingdao Marine Science and Technology Center, Qingdao, 266100, China.*

[3]*College of Chemistry and Chemical Engineering, Ocean University of China, Qingdao, 266100, China.*

[4]*Institute for Advanced Ocean Study, Physical Oceanography Laboratory, Ocean University of China, Qingdao, 266100, China.*

[5]*Department of Earth, Planetary and Space Sciences, University of California, Los Angeles, California, 90095, USA.*

[6]*School of Biological Sciences, University of East Anglia, Norwich Research Park, Norwich, NR4 7TJ, UK.*

Correspondence: Guang-Chao Zhuang (zgc@ouc.edu.cn); Hong-Hai Zhang (honghaizhang@ouc.edu.cn); College of Chemistry and Chemical Engineering, Ocean University of China, 238 Songling Road, Qingdao, 266100, China.

Content: Fig. S1-S5; Table S1-S2

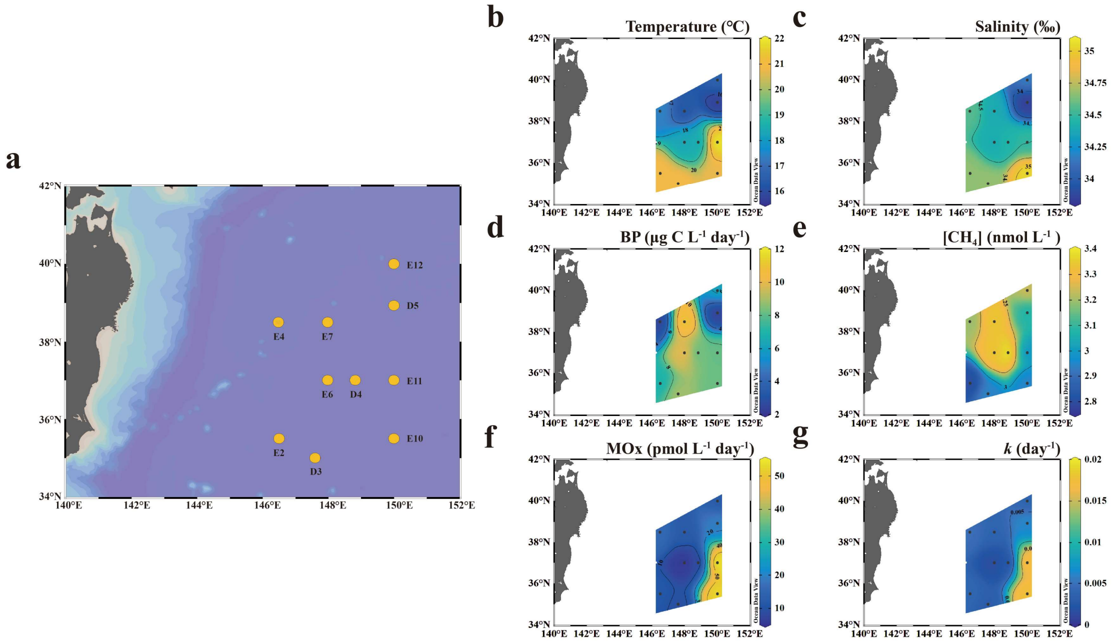

Fig. S1: The methane oxidation rates of surface seawater at the KOE region. a Sampling sites during the June 2021 cruise**. b-g** The spatial distribution of temperature, salinity, bacterial production rates (BP), methane concentration ([CH_4]), methane oxidation rates (MOx) and methane oxidation rates constant (*k*).

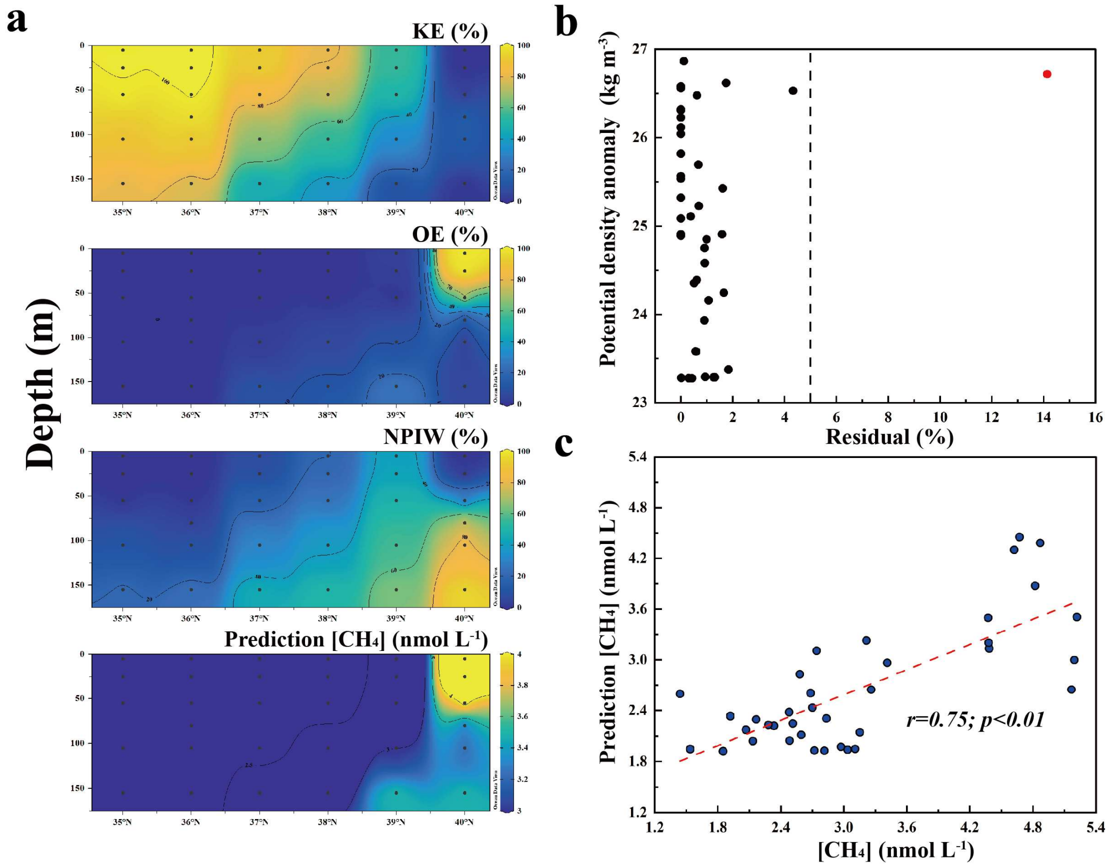

Fig. S2: The optimum multiparameter (OMP) analysis. **a** The contribution of different water mass to the spatial distribution of methane at the P section: Kuroshio Extension (KE), Oyashio Extension (OE), North Pacific Intermediate Water (NPIW). **b** Plot of the mass balance residuals computed through OMP analysis versus potential density anomaly. With the exception of one data point with a residual higher than 14% (red dot), all other data points have residuals lower than 5%. **c** Plot of methane concentration predicted by OMP model (*y* axis) versus observed methane concentration (*x* axis). The red dashed line represents a linear fitting of all the data points.

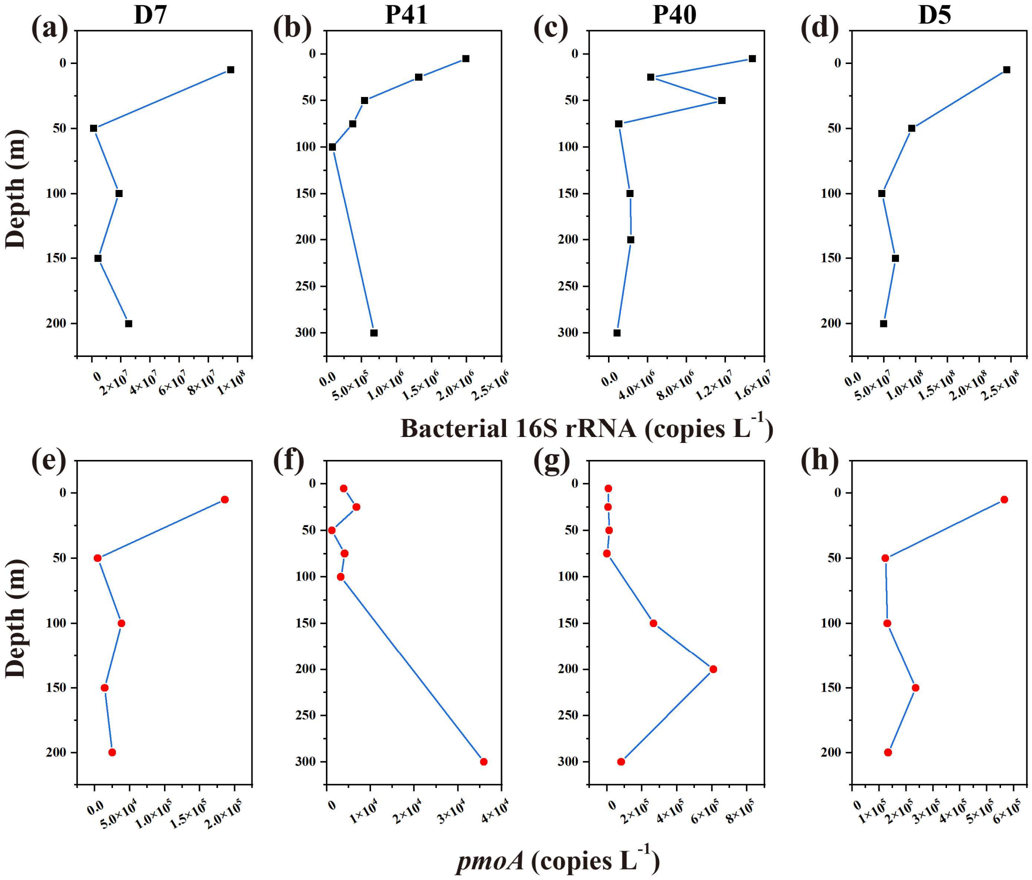

Fig. S3: The abundance of bacterial 16S rRNA and *pmoA* genes above 300 m water column at the KOE region in June 2022. Panels **a** and **e** show data for the D7 site (42° N); **b** and **f** for the P41 site (41° N); **c** and **g** for the P40 site (40° N); and **d** and **h** for the D5 site (39° N).

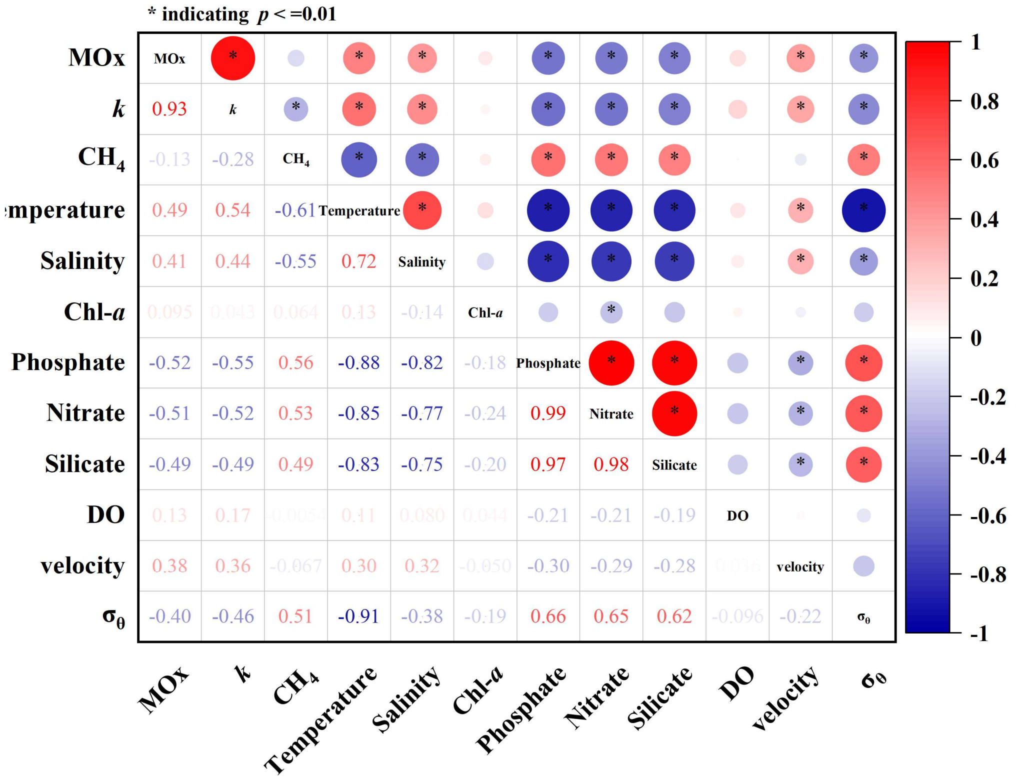

Fig. S4. The correlation analysis between MOx and other environmental factors in the KOE region during the July 2022 cruise. '*' indicating *p* < =0.01.

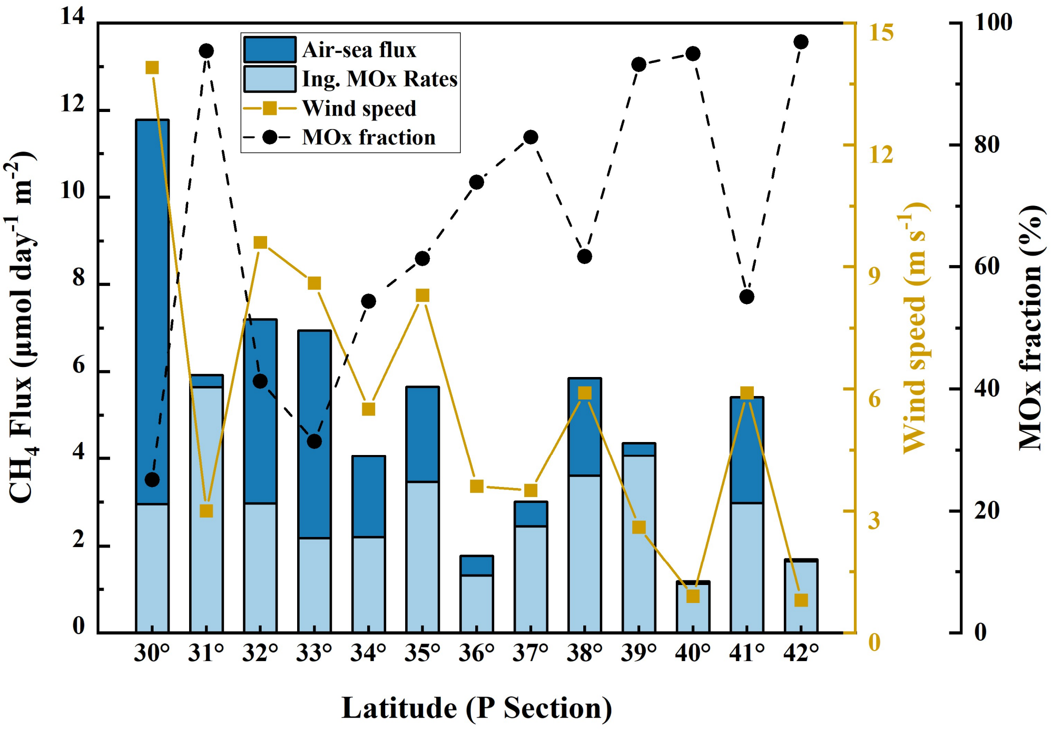

Fig. S5: Depth-integrated methane oxidation rates (light blue column), air-sea flux (dark blue column), MOx fraction (brown square) and wind speeds (black dot) during the July 2022 cruise. MOx fraction = 100 × Ing. MOx rates/(Ing. MOx rates + air-sea flux). The depth range for integration was from the mixed layer to the methane maximum layer. Wind speed was obtained from the shipboard meteorology observation system at 10 m above the sea surface.

Table S1. Source water specific properties considered in the OMP method

Water type[a]	Temperture (°C)	Salinity (‰)	DO (μmol kg^{-1})	Phosphate (μmol L^{-1})	Nitrate (μmol L^{-1})	Silicate (μmol L^{-1})
OE	14.53	33.46	237	0.31	2.38	5.31
KE	18.11	34.8	202.2	0.303	3.4	3.3
NPIW	3.94	33.82	112.1	0.98	12.45	19.92

[a]OE, Oyashio Extension; KE, Kuroshio Extension; NPIW, North Pacific Intermediate Water.

Table S2. Summary of linear regression statistics and associated LMP value in percent contribution to the overall R^2 of the regression[a].

	Coefficients	Estimate	Standard error	t value	Pr(>|t|)[b]	LMP (%)
pCO_2	Intercept	569.23	81.96	6.94	***	
	Temperature	1.87	0.22	8.49	***	40.38
	Salinity	–2.73	2.24	–1.22		24.87
	Oxygen saturation	–1.62	0.23	–6.91	***	34.75
$[CH_4]$	Intercept	8.81	0.54	16.27	***	
	Temperature	–0.037	0.0014	–24.90	***	42.26
	Salinity	–0.175	0.0148	–11.78	***	33.89
	Oxygen saturation	0.0049	0.0015	3.13	**	23.85

[a]Calculations were done by the R package "relaimpo" (Grömping 2006).

[b]Significance codes: '***' indicating $p < 0.001$, '**' indicating $0.001 <= p < 0.01$, '*' indicating $0.01 <= p < 0.05$.
